# Immunosenescence and age-related viral diseases

**DOI:** 10.1007/s11427-013-4478-0

**Published:** 2013-05-01

**Authors:** YongChao Ma, Min Fang

**Affiliations:** CAS Key Laboratory of Pathogenic Microbiology and Immunology, Institute of Microbiology, Chinese Academy of Sciences, Beijing, 100101 China

**Keywords:** immunosenescence, physiologic ageing, infectious disease

## Abstract

Immunosenescence is described as a decline in the normal functioning of the immune system associated with physiologic ageing. Immunosenescence contributes to reduced efficacy to vaccination and increased susceptibility to infectious diseases in the elderly. Extensive studies of laboratory animal models of ageing or donor lymphocyte analysis have identified changes in immunity caused by the ageing process. Most of these studies have identified phenotypic and functional changes in innate and adaptive immunity. However, it is unclear which of these defects are critical for impaired immune defense against infection. This review describes the changes that occur in innate and adaptive immunity with ageing and some age-related viral diseases where defects in a key component of immunity contribute to the high mortality rate in mouse models of ageing.

## 1 Ageing and immunosenescence

The human lifespan has significantly increased in the developed world over past decades because of socioeconomic development and medical care improvement. The global population is ageing and the proportion of elderly individuals aged 60 years and older is expected to reach 21% of the whole population by 2050 [1]. This presents a major challenge to public health services as the elderly are a vulnerable population to emerging infectious diseases, such as pandemic influenza (H5N1), West Nile virus (WNV) and severe acute respiratory syndrome (SARS) [2–4]. Moreover, immunosenescence renders vaccination less effective in the elderly. For example, the yearly influenza vaccine is only 29%–46% efficacious in persons aged ⩾75 years and 41%–58% in persons 60–74 years old [5] compared to 70%–90% in young adults [4]. Additionally, reduced immune responses have been observed in elderly individuals vaccinated against tetanus, hepatitis and *Streptococcus pneumonia* [2]. Thus, there is an urgent need to develop strategies for improving vaccination efficacy and therapies to strengthen immunity in the elderly. However, to achieve this goal, a thorough understanding of the mechanisms underlying the age-related decline in immunity is needed.

## 2 Ageing and the adaptive immune system

Adaptive immunity includes B and T lymphocytes that respond to challenges with a high degree of specificity and memory. The competency of the adaptive immune function decreases with age. Both T cell mediated cellular immunity and B cell mediated humoral immunity are involved in immunosenescence.

### 2.1 Effect of ageing on T cell immunity

The age-related decline in T cell immunity is mainly characterized by a decrease in numbers of naïve T cells [6,7]

and the expansion of memory T cells, especially late-stage differentiated $CD8^+$ cells. These result in reduced T cell TCR diversity and function [8–10]. Another feature of the T cell compartment in ageing humans or mice is the accumulation of large oligoclonal expansions of $CD8^+$ T cells that are often cytomegalovirus (CMV)-specific [11–13]. Through longitudinal studies, Pawelec et al. [14] found that CMV seropositivity has a direct relation to immune risk phenotype (IRP). The IRP is characterized by decreasing naïve $CD4^+$ cell numbers, an inverted ratio of CD4/CD8 cells and $CD8^+$ T cell repertoire (TCR) disturbance [15–17]. About 60%–100% of the human population is persistently or latently infected with CMV, and chronic infection by CMV may accelerate ageing of the immune system and lead to a high levels of chronic subclinical inflammation [18]. CMV-specific $CD8^+$ T cell expansion, resulting in a decrease of peripheral naïve $CD8^+$ T cells and TCR diversity contraction, causes weak responses to new infectious pathogens [15,16]. Whether other viruses, such as Epstein-Barr virus (EBV), herpesviruses (human herpesvirus, HHV) or polyomaviruses (BK virus, JC virus) may also contribute to accelerated immunosenescence in the aged population is still not fully understood [19–21].

Another factor that contributes to immunosenescence of the T cell compartment is age-associated thymic involution. The decline in thymus function causes a dramatic decrease in naïve T cell production in aged individuals [22,23]. This may be a good interpretation for immunosenescence, however, it cannot explain the fact that the total number of T lymphocytes is maintained quite well by homeostasis in healthy people [24,25]. Functional and phenotypic analysis indicated that other factors probably influence the process of age-related immunosenescence. Persistent or latent virus infection or other environmental factors may be "boosting elements", but further studies are needed to investigate the impact of environmental factors on the ageing process.

### 2.2 Effect of ageing on B cell mediated humoral immunity

Age-associated immune dysfunction also occurs in B cell compartments, which results in impaired humoral immune functions [26,27]. However, both naïve and memory B cells are decreased with ageing [28,29], in contrast with the T cell compartments. Although the percentage and number of human B cell precursors in the bone marrow do not decline significantly during ageing [30], the total level of mature human B cells in peripheral blood significantly decreases with age [31–33]. Like T cell compartments, antigen-experienced memory cells are most prevalent in the peripheral B cell pool, resulting in limited B cell clonal expansion and repertoire diversity limitation [34,35]. Based on different antibody isotypes, human memory B cells can be subdivided into two distinct types: IgM memory ($IgG^-IgA^-CD27^+$) and switch memory ($IgG^+IgA^+$) B cells [36]. The absolute number of IgM memory B cells significantly decreases as a result of the decline of total B cell numbers. However, the percentage remains unchanged. However, the percentage and number of switch memory B cells decreases during ageing [37]. Functional analysis studies have demonstrated that both IgM memory B cells and switch memory B cells are impaired in the elderly, which helps to explain why vaccines have poor effects against infections in aged people [31,38]. Above all, antibodies secreted from B cells are also influenced by ageing. Johnson et al. [39] demonstrated that antibody affinity in aged individuals is lower than in young people, which also contributes to reduced protection from vaccination.

Both humoral and cellular immune systems are affected in the ageing process, and most importantly, they are influenced reciprocally. As an integral component of the immune system, lymph nodes are the terminal point for collecting antigens from peripheral tissues and recruiting naïve lymphocytes from the blood, providing the microenvironment for primary and secondary immune responses. Therefore, the lymph node is the perfect place for studying T cell and B cell interactions. Lazuardi and colleagues demonstrated that interactions between T and B cells was impaired in aged people, leading to reduced B cell expansion and differentiation in response to antigens [40].

## 3 Ageing and innate immunity

The innate immune system includes a diverse group of cell types including neutrophils, natural killer cells (NK), natural killer T cells (NKT), monocytes/macrophages and dendritic cells (DCs). The innate immune system, which serves as an immunological sentinel defense against microbial pathogens, is also affected at multiple levels during the ageing process [41,42], but the effects and mechanisms remain incompletely understood, particularly in humans. Here we discuss the recent findings in age-associated changes in NK cells.

NK cells are an important component in innate immunity, play a critical role in host defense against invading pathogens and malignant transformation through rapid cytolytic activity or direct production of cytokines, such as interferon (IFN)-γ and tumor necrosis factor (TNF)-α [43,44]. NK cells are derived from bone marrow. In humans, NK cells comprise about 15% of all blood lymphocytes and are defined phenotypically by their expression of CD56 surface antigen and lack of expression of CD3 [43,45]. According to the cell surface density of CD56 and CD16 expression (FcγRIII), human NK cells can be divided into two distinct subsets, the majority (~90% in blood) of human NK cells are $CD56^{dim}CD16^+$, and a minor population (~10% in blood) is $CD56^{bright}CD16^-$ [45]. Early functional studies revealed that $CD56^{bright}$ cells have the ability to produce abundant cytokines and have a high cytotoxic function, whereas

$CD56^{dim}$ cells may function as efficient effectors of natural and antibody-dependent target cell lysis [46,47]. However, when resting NK cells were activated with interleukin (IL)-2 or IL-12 *in vitro*, $CD56^{bright}$ and $CD56^{dim}$ cells exhibited similar levels of cytotoxic ability [48,49].

NK cell immune functions and cellular phenotype are also influenced during ageing. Age-related changes in NK cell functionality are controversial. Previous studies focused on the cytotoxicity and numbers of NK cells in elderly people have reported different and contradictory conclusions. Recent studies have demonstrated that the number of NK cells is stable or increases during ageing [50–54]. In addition, the NK subpopulations are also dynamically changed during ageing. The absolute number of $CD56^{bright}$ $CD16^{-}$ cells declines with age and is associated with an increase in the $CD56^{dim}CD16^{+}$ population (highly mature cytotoxic subpopulation) [55]. This change results in increased NK cytotoxicity in elderly individuals, but from a per-cell based activity analysis, cytotoxic activation decreases with age [55–58]. The mechanisms of NK cell cytotoxic defects remain unknown.

Mature NK cells normally remain quiescent until virus infection or other stress. They then rapidly proliferate initially in a nonspecific mode. The cytotoxicity of activated NK cells is regulated by the balance of signals from their own activating and inhibitory receptors [59]. To date, three receptor families have been characterized, including killer cell immunoglobulin-like receptors (KIRs), natural cytotoxicity receptors (NCRs), and C-type lectins [59–61]. Recent studies have begun to elucidate age-associated changes of NK cell receptors. Lutz et al. demonstrated that the expression of KIRs is increased in the elderly population and this phenomenon is also observed in T-cell related receptor expression [62–64]. In contrast, Almeida-Oliveira found no age-related changes in overall KIR expression on NK cells, but demonstrated an increase in KIR expression in the $CD56^{bright}$ subset of elderly subjects instead [65]. Their results are similar to a previous report [66]. Moreover, they also found the expression of C-type lectin CD94 and activating NCRs (NKp30, NKp46) decreased significantly in older adults compared to younger adults [65].

Once activated, NK cells exhibit direct cytolytic activity on infected cells and release pro-inflammatory cytokines and chemokines that contribute to the adaptive Th1 immune response [67]. Some limited studies have focused on the effect ageing has on the production of cytokines and the regulatory role of NK cells on adaptive immunity. Previous studies indicated that cytokines and chemokines produced by NK cells in rodents decreased with advancing age. It was observed that IL-2-induced IFN-γ production and IL-2- or IL-12-induced production of chemokines such as macrophage inflammatory protein 1a (MIP-1a) and IL-8 were decreased in NK cells from aged individuals [68,69]. Mocchegiani et al. [70] showed that NK cell production of IFN-γ, TNF-α, IL-2 and IL-12 was decreased in elderly individuals and might contribute to T cell deficits associated with ageing. However, in a recent study, IFN-γ production upon NK cell activation was shown to be significantly higher in the aged, particularly in subjects older than 85 years of age compared to younger adults [71]. Moreover, they found IFN-γ-expressing NK cells correlated positively with the serum content of the –SH groups in seniors. Their findings indicated that activation of NK cells at an advanced age might be related to the oxidative and inflammatory status of the elderly.

Many factors may influence the different results in these studies regarding NK cell activity, including (i) the criteria of selected subjects (age, sex, race, etc.); (ii) the subjects' basic health and nutrition status; (iii) potential infection to virus or other pathogens; and (iv) chronic inflammation. At present, there is no standard criterion of senescence and it seems to vary with the development of the economy and subjects' physical quality. Some studies indicated that NK cell activity and cytotoxicity could be modulated by external agents such as zinc [70,72]. Recently, "inflamm-ageing" has been described, where healthy ageing individuals have an active inflammatory state, with high levels of IL-6, IL-1β and tumor necrosis factor-α (TNF-α) [73]. Whether the elevation of these cytokines can regulate NK cell activity remains unknown. Clearly, we are still at the beginning of understanding the complicated changes in NK cell functions with ageing and further studies are needed to identify the mechanisms underlying these changes.

## 4 Specific defects in the immune system and age-related viral diseases

Although the defects in both the innate and adaptive immune system contribute to immunosenescence, studies have also shown that it is significantly heterogeneous in individuals. Moreover, the primary immune response is also different in the same individuals when infected with different pathogens. Experimental mice models have provided a good research platform to study age-related susceptibility to certain infectious diseases.

### 4.1 T cell defects and West Nile virus infection

West Nile virus (WNV) is the main cause of encephalitis in the West Nile region. The natural reservoirs for WNV are mosquitoes and birds, but it can also infect humans and other mammals [74]. There have been outbreaks and pandemics in North America and Africa and other countries in recent years [75,76]. The mortality rate in the infected population is about 10%. WNV disease is an age-related disease. The prevalence and mortality gradually increase during ageing, and the median age of death caused by WNV is 78 years [77]. There is no protective vaccine or efficient antiviral treatment available for WNV infection. Studies have indicated that the innate immune system and T/B-cell mediated immunity are involved in the control of WNV infection. B cell-deficient mice develop high viral titers in the blood [78]. However, T cell-mediated immunity seems to play a dominant role in the control of WNV infection. T-cell defects significantly increase the lethality of WNV infection, and the survivors take many years to fully recover physical, functional and cognitive ability [79,80] .

Using an aged mouse model, Brien et al. [81] showed that defects in T cells play a key role in age-related vulnerability to WNV infection. Aged mice infected with WNV had a high mortality rate due to uncontrolled virus spread to the brain because of CD8 and CD4 T-cell functional defects. T cell responses in aged mice following WNV infection were weak demonstrated by insufficient numbers of ineffective effector T cells accumulating in the brains of WNV-infected old mice. Both CD4 and CD8 T cells exhibited profound quantitative and qualitative defects, but these defects did not extend to all aspects of antigen recognition and activation. Furthermore, it seems that the ability of T cells to migrate into the mouse brain was not diminished, but that the infiltrating T cells had a diminished effector function [81]. Adoptive transfer of adult CD4 or CD8 T cells significantly protected aged mice against lethal WNV infection. Collectively, specific age-related defects in T cell immunity, affecting both CD8 and CD4 T cells, are the main reason for the increased susceptibility to WNV infection in the mouse model of ageing.

### 4.2 NK cells defects and the mousepox model

Mousepox is a viral disease caused by a specific mouse orthopoxvirus (OPV), called ectromelia virus (ECTV). ECTV is genetically very similar to the human pathogen variola virus (the agent of smallpox), and monkeypox virus (the agent of monkeypox). Monkeypox is a serious endemic disease in central Africa and a recent outbreak occurred in the USA [82,83]. Although all mouse strains can be infected with ECTV, the outcome of the infection following footpad inoculation varies. Some sensitive strains such as BALB/c develop mousepox and endure a very high mortality rate during the first two weeks post-infection (PI) [84,85], while other strains such as C57BL/6 (B6) control the infection without visible symptoms. The resistance of B6 mice to disease is not due to an inherent decreased ability of the virus to replicate in this strain but to the combined action of the innate and adaptive immune systems. Deficiencies in innate immune components caused by NK cell or macrophage depletion, or deficiencies in adaptive immune components such as in CD8 T cell knockout mice, B cell knockout mice or severely immunodeficient (recombinase activating gene 1 knockout mice) mice in resistant B6 background, all readily succumb to mousepox [86–90].

NK cells play an important role in controlling primary mousepox. Recently, we identified the mechanisms of NK cell mediated resistance to mousepox. After footpad ECTV infection, NK cells are activated and migrate to the local draining lymph nodes (D-LN), controlling early virus dissemination to the central organs by direct cytolytic function and by secreting anti-viral cytokines. NK cells responses also augment antiviral T cell responses [87]. Moreover, we also showed that NK cell activating receptor CD94-NKG2E can specifically recognize ECTV-infected cells, and another NK cell activating receptor NKG2D functions as a costimulator [91].

We observed that B6 mice gradually lose their natural resistance to mousepox as they age, and that resistance starts to wane at a relatively early age (~6 months) and is completely lost when mice reach 14 months of age. Surprisingly, the main reason for the loss of resistance is not because of intrinsically defective T cell responses. Instead, the primary reason is because of defects in NK cell responses in the aged mice. We observed a decrease in the number of total and mature NK cells in the blood of aged mice [92]. In addition, after ECTV infection, mature NK cells in aged mice have an intrinsic defect in migration to the D-LN to prevent systemic virus spread. In our previous work, we demonstrated that D-LN is a major site for the prevention of viral spread [93]. Furthermore, lymph nodes are an important site for the initiation of immune responses, and where NK cells interact with other immune cells such as DCs and T cells, to regulate adaptive immune functions [94]. Therefore, our results demonstrate that NK cells from aged mice have defects in their ability to migrate to the D-LN after ECTV infection, which results in increased early virus replication and spread as well as susceptibility to an acute viral disease. A model of this is shown in Figure 1.

In contrast to the T cell defects observed in age-dependent susceptibility to WNV infection, NK cell defects directly result in the age-related susceptibility to mousepox. However, in both models, T cells and NK cells show impaired qualitative and quantitative functions. In the WNV model, the ability of T cells to migrate to the brain seems not to be defective; whereas in the mousepox model, mature NK cells have intrinsic defects in their migration to D-LNs. We further show that decreased CD62L expression on mature NK cells from aged mice might contribute to impaired migration. However, in both cases, the mechanisms underlying the functional defects are unknown. A comparison of the two models is summarized in Table 1.

## 5 Conclusion

Clearly, immunosenescence affects both innate and adaptive immunity. The key components of the ageing immune systems for protection against infectious diseases may vary as a result of different viruses, the infection route and dose. Although significant progress has been made in recent years, results from different experimental models and different

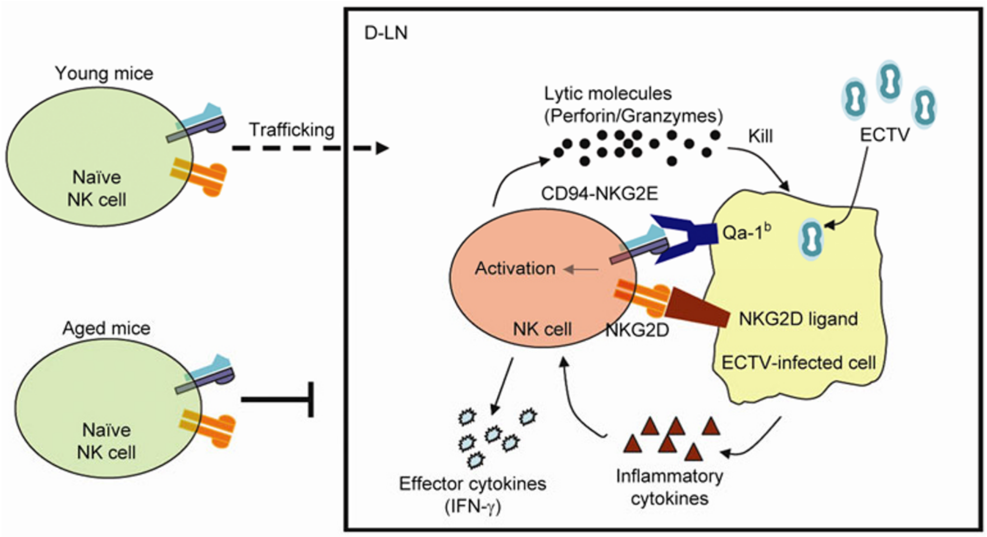

**Figure 1** Impaired NK cell responses result in a high mortality rate in aged mice upon ECTV infection. Following Ectromelia virus (ECTV) infection in young mice, natural killer (NK) cells, probably activated by cytokines, migrate to the D-LN and kill ECTV-infected cells via CD94-NKG2E that specifically recognize infected targets and NKG2D that acts as a costimulator. After ECTV infection in aged mice, NK cells fail to migrate to the D-LN to prevent early virus dissemination, which directly causes the death of aged mice.

**Table 1** Comparisons between aged-related susceptibility to WNV and ECTV infection[a)]

| Model | West Nile virus | Ectromelia virus |
|---|---|---|
| Immune system defects | T cells | NK cells |
| Functional impairments | Qualitative and quantitative | Qualitative and quantitative |
| Migration ability | Normal | Defect |
| Cause of death | Meningoencephalitis | Liver necrosis |

a) WNV, West Nile virus; ECTV, Ectromelia virus; NK, natural killer cells.

subjects are not always consistent, and can show opposing results. Understanding how and why immune responsiveness changes in humans as they age is essential for developing strategies to prevent or restore impaired immunity and assure healthy longevity. We are still at the beginning of unraveling the mystery of immunosenescence. The knowledge we gain from further in depth studies will pave the way for rational interventions to maintain or restore appropriate immune function in the elderly.

1 Aspinall R, Del Giudice G, Effros R B, et al. Challenges for vaccination in the elderly. Immun Ageing, 2007, 4: 9
2 Ongradi J, Stercz B, Kovesdi V, et al. Immunosenescence and vaccination of the elderly II. New strategies to restore age-related immune impairment. Acta Microbiol Immunol Hung, 2009, 56: 301–312
3 Andrews N P, Fujii H, Goronzy J J, et al. Telomeres and immunological diseases of aging. Gerontology, 2010, 56: 390–403
4 Bernstein E, Kaye D, Abrutyn E, et al. Immune response to influenza vaccination in a large healthy elderly population. Vaccine, 1999, 17: 82–94
5 Weinberger B, Herndler-Brandstetter D, Schwanninger A, et al. Biology of immune responses to vaccines in elderly persons. Clin Infect Dis, 2008, 46: 1078–1084
6 Moon J J, Chu H H, Pepper M, et al. Naive CD4(+) T cell frequency varies for different epitopes and predicts repertoire diversity and response magnitude. Immunity, 2007, 27: 203–213
7 Naylor K, Li G, Vallejo A N, et al. The influence of age on T cell generation and TCR diversity. J Immunol, 2005, 174: 7446–7452
8 Leng S X. Role of chronic cytomegalovirus infection in T-cell immunosenescence and frailty: more questions than answers. J Am Geriatr Soc, 2011, 59: 2363–2365
9 Voehringer D, Koschella M, Pircher H. Lack of proliferative capacity of human effector and memory T cells expressing killer cell lectinlike receptor G1 (KLRG1). Blood, 2002, 100: 3698–3702
10 LeMaoult J, Messaoudi I, Manavalan J S, et al. Age-related dysregulation in CD8 T cell homeostasis: kinetics of a diversity loss. J Immunol, 2000, 165: 2367–2373
11 Yager E J, Ahmed M, Lanzer K, et al. Age-associated decline in T cell repertoire diversity leads to holes in the repertoire and impaired immunity to influenza virus. J Exp Med, 2008, 205: 711–723
12 Pawelec G, Akbar A, Beverley P, et al. Immunosenescence and Cytomegalovirus: where do we stand after a decade? Immun Ageing, 2010, 7: 13
13 Pawelec G, Akbar A, Caruso C, et al. Is immunosenescence infectious? Trends Immunol, 2004, 25: 406–410
14 Vasto S, Colonna-Romano G, Larbi A, et al. Role of persistent CMV infection in configuring T cell immunity in the elderly. Immun Ageing, 2007, 4: 2
15 Barrett L, Fowke K R, Grant M D. Cytomegalovirus, aging, and HIV: a perfect storm. AIDS Rev, 2012, 14: 159–167
16 Akbar A N, Fletcher J M. Memory T cell homeostasis and senescence during aging. Curr Opin Immunol, 2005, 17: 480–485
17 Almanzar G, Schwaiger S, Jenewein B, et al. Long-term cytomegalovirus infection leads to significant changes in the composition of the CD8$^{+}$ T-cell repertoire, which may be the basis for an imbalance in the cytokine production profile in elderly persons. J Virol, 2005, 79: 3675–3683
18 Khan N, Hislop A, Gudgeon N, et al. Herpesvirus-specific CD8 T cell immunity in old age: cytomegalovirus impairs the response to a coresident EBV infection. J Immunol, 2004, 173: 7481–7489
19 Zhong S, Zheng H Y, Suzuki M, et al. Age-related urinary excretion of BK polyomavirus by nonimmunocompromised individuals. J Clin Microbiol, 2007, 45: 193–198

20 Decman V, Laidlaw B J, Dimenna L J, et al. Cell-intrinsic defects in the proliferative response of antiviral memory CD8 T cells in aged mice upon secondary infection. J Immunol, 2010, 184: 5151–5159

21 Naumova E, Ivanova M, Pawelec G. Immunogenetics of ageing. Int J Immunogenet, 2011, 38: 373–381

22 Vallejo A N. Age-dependent alterations of the T cell repertoire and functional diversity of T cells of the aged. Immunol Res, 2006, 36: 221–228

23 Aw D, Palmer D B. The origin and implication of thymic involution. Aging Dis, 2011, 2: 437–443

24 Goronzy J J, Weyand C M. T cell development and receptor diversity during aging. Curr Opin Immunol, 2005, 17: 468–475

25 Effros R B, Dagarag M, Spaulding C, et al. The role of $CD8^+$ T-cell replicative senescence in human aging. Immunol Rev, 2005, 205: 147–157

26 Frasca D, Diaz A, Romero M, et al. Age effects on B cells and humoral immunity in humans. Ageing Res Rev, 2011, 10: 330–335

27 Frasca D, Blomberg B B. Effects of aging on B cell function. Curr Opin Immunol, 2009, 21: 425–430

28 Paganelli R, Quinti I, Fagiolo U, et al. Changes in circulating B cells and immunoglobulin classes and subclasses in a healthy aged population. Clin Exp Immunol, 1992, 90: 351–354

29 Mehr R, Melamed D. Reversing B cell aging. Aging, 2011, 3: 438–443

30 LeMaoult J, Szabo P, Weksler M E. Effect of age on humoral immunity, selection of the B-cell repertoire and B-cell development. Immunol Rev, 1997, 160: 115–126

31 Shi Y, Yamazaki T, Okubo Y, et al. Regulation of aged humoral immune defense against pneumococcal bacteria by IgM memory B cell. J Immunol, 2005, 175: 3262–3267

32 Frasca D, Riley R L, Blomberg B B. Humoral immune response and B-cell functions including immunoglobulin class switch are downregulated in aged mice and humans. Semin Immunol, 2005, 17: 378–384

33 Van der Put E, Sherwood E M, Blomberg B B, et al. Aged mice exhibit distinct B cell precursor phenotypes differing in activation, proliferation and apoptosis. Exp Gerontol, 2003, 38: 1137–1147

34 LeBien T W, Tedder T F. B lymphocytes: how they develop and function. Blood, 2008, 112: 1570–1580

35 Bernasconi N L, Traggiai E, Lanzavecchia A. Maintenance of serological memory by polyclonal activation of human memory B cells. Science, 2002, 298: 2199–2202

36 Seifert M, Kuppers R. Molecular footprints of a germinal center derivation of human $IgM^+(IgD^+)CD27^+$ B cells and the dynamics of memory B cell generation. J Exp Med, 2009, 206: 2659–2669

37 Macallan D C, Wallace D L, Zhang Y, et al. B-cell kinetics in humans: rapid turnover of peripheral blood memory cells. Blood, 2005, 105: 3633–3640

38 Frasca D, Diaz A, Romero M, et al. Intrinsic defects in B cell response to seasonal influenza vaccination in elderly humans. Vaccine, 2010, 28: 8077–8084

39 Johnson S A, Cambier J C. Ageing, autoimmunity and arthritis: senescence of the B cell compartment-implications for humoral immunity. Arthritis Res Ther, 2004, 6: 131–139

40 Lazuardi L, Jenewein B, Wolf A M, et al. Age-related loss of naive T cells and dysregulation of T-cell/B-cell interactions in human lymph nodes. Immunology, 2005, 114: 37–43

41 Ginaldi L, De Martinis M, D'Ostilio A, et al. The immune system in the elderly: III. Innate immunity. Immunol Res, 1999, 20: 117–126

42 Panda A, Arjona A, Sapey E, et al. Human innate immunosenescence: causes and consequences for immunity in old age. Trends Immunol, 2009, 30: 325–333

43 Cooper M A, Fehniger T A, Caligiuri M A. The biology of human natural killer-cell subsets. Trends Immunol, 2001, 22: 633–640

44 Lanier L L. NK cell recognition. Annu Rev Immunol, 2005, 23: 225–274

45 Robertson M J, Ritz J. Biology and clinical relevance of human natural killer cells. Blood, 1990, 76: 2421–2438

46 Lanier L L, Testi R, Bindl J, et al. Identity of Leu-19 (CD56) leukocyte differentiation antigen and neural cell adhesion molecule. J Exp Med, 1989, 169: 2233–2238

47 Campbell J J, Qin S, Unutmaz D, et al. Unique subpopulations of $CD56^+$ NK and NK-T peripheral blood lymphocytes identified by chemokine receptor expression repertoire. J Immunol, 2001, 166: 6477–6482

48 Nagler A, Lanier L L, Phillips J H. Constitutive expression of high affinity interleukin 2 receptors on human CD16-natural killer cells *in vivo*. J Exp Med, 1990, 171: 1527–1533

49 Robertson M J, Soiffer R J, Wolf S F, et al. Response of human natural killer (NK) cells to NK cell stimulatory factor (NKSF): cytolytic activity and proliferation of NK cells are differentially regulated by NKSF. J Exp Med, 1992, 175: 779–788

50 Sansoni P, Cossarizza A, Brianti V, et al. Lymphocyte subsets and natural killer cell activity in healthy old people and centenarians. Blood, 1993, 82: 2767–2773

51 Thompson J S, Wekstein D R, Rhoades J L, et al. The immune status of healthy centenarians. J Am Geriatr Soc, 1984, 32: 274–281

52 Burkle A, Caselli G, Franceschi C, et al. Pathophysiology of ageing, longevity and age related diseases. Immun Ageing, 2007, 4: 4

53 Miyaji C, Watanabe H, Toma H, et al. Functional alteration of granulocytes, NK cells, and natural killer T cells in centenarians. Hum Immunol, 2000, 61: 908–916

54 Vitale M, Zamai L, Neri L M, et al. The impairment of natural killer function in the healthy aged is due to a postbinding deficient mechanism. Cell Immunol, 1992, 145: 1–10

55 Solana R, Tarazona R, Gayoso I, et al. Innate immunosenescence: effect of aging on cells and receptors of the innate immune system in humans. Semin Immunol, 2012, 24: 331–341

56 Borrego F, Alonso M C, Galiani M D, et al. NK phenotypic markers and IL2 response in NK cells from elderly people. Exp Gerontol, 1999, 34: 253–265

57 Ogata K, Yokose N, Tamura H, et al. Natural killer cells in the late decades of human life. Clin Immunol Immunopathol, 1997, 84: 269–275

58 Simpson R J, Lowder T W, Spielmann G, et al. Exercise and the aging immune system. Ageing Res Rev, 2012, 11: 404–420

59 Farag S S, Fehniger T, Ruggeri L, et al. Natural killer cells: biology and application in stem-cell transplantation. Cytotherapy, 2002, 4: 445–446

60 Sconocchia G, Lau M, Provenzano M, et al. The antileukemia effect of HLA-matched NK and NK-T cells in chronic myelogenous leukemia involves NKG2D-target-cell interactions. Blood, 2005, 106: 3666–3672

61 Verheyden S, Ferrone S, Mulder A, et al. Role of the inhibitory KIR ligand HLA-Bw4 and HLA-C expression levels in the recognition of leukemic cells by Natural Killer cells. Cancer Immunol Immunother, 2009, 58: 855–865

62 Lutz C T, Moore M B, Bradley S, et al. Reciprocal age related change in natural killer cell receptors for MHC class I. Mech Ageing Dev, 2005, 126: 722–731

63 Lemster B H, Michel J J, Montag D T, et al. Induction of CD56 and TCR-independent activation of T cells with aging. J Immunol, 2008, 180: 1979–1990

64 Abedin S, Michel J J, Lemster B, et al. Diversity of NKR expression in aging T cells and in T cells of the aged: the new frontier into the exploration of protective immunity in the elderly. Exp Gerontol, 2005, 40: 537–548

65 Almeida-Oliveira A, Smith-Carvalho M, Porto L C, et al. Age-related changes in natural killer cell receptors from childhood through old age. Hum Immunol, 2011, 72: 319–329

66 Le Garff-Tavernier M, Beziat V, Decocq J, et al. Human NK cells display major phenotypic and functional changes over the life span. Aging cell, 2010, 9: 527–535

67 Solana R, Mariani E. NK and NK/T cells in human senescence. Vaccine, 2000, 18: 1613–1620

68 Krishnaraj R, Bhooma T. Cytokine sensitivity of human NK cells during immunosenescence. 2. IL2-induced interferon gamma secretion, Immunol Lett, 1996, 50: 59–63

69 Mariani E, Pulsatelli L, Neri S, et al. RANTES and MIP-1alpha production by T lymphocytes, monocytes and NK cells from nonagenarian subjects. Exp Gerontol, 2002, 37: 219–226

70 Mocchegiani E, Giacconi R, Cipriano C, et al. The variations during the circadian cycle of liver CD1d-unrestricted NK1.1+TCR gamma/delta+ cells lead to successful ageing. Role of metallothionein/IL-6/gp130/PARP-1 interplay in very old mice. Exp Gerontol, 2004, 39: 775–788

71 Kaszubowska L, Kaczor J J, Hak L, et al. Sensitivity of natural killer cells to activation in the process of ageing is related to the oxidative and inflammatory status of the elderly. J Physiol Pharmacol, 2011, 62: 101–109

72 Mocchegiani E, Muzzioli M, Giacconi R, et al. Metallothioneins/PARP-1/IL-6 interplay on natural killer cell activity in elderly: parallelism with nonagenarians and old infected humans. Effect of zinc supply. Mech Ageing Dev, 2003, 124: 459–468

73 Franceschi C, Bonafe M, Valensin S, et al. Inflamm-aging. An evolutionary perspective on immunosenescence. Ann N Y Acad Sci, 2000, 908: 244–254

74 Rossi S L, Ross T M, Evans J D. West Nile virus. Clin Lab Med, 2010, 30: 47–65

75 Komar N, Clark G G. West Nile virus activity in Latin America and the Caribbean. Rev Panam Salud Publica, 2006, 19: 112–117

76 Lanciotti R S, Ebel G D, Deubel V, et al. Complete genome sequences and phylogenetic analysis of West Nile virus strains isolated from the United States, Europe, and the Middle East. Virology, 2002, 298: 96–105

77 Murray K, Baraniuk S, Resnick M, et al. Risk factors for encephalitis and death from West Nile virus infection. Epidemiol Infect, 2006, 134: 1325–1332

78 Diamond M S, Shrestha B, Marri A, et al. B cells and antibody play critical roles in the immediate defense of disseminated infection by West Nile encephalitis virus. J Virol, 2003, 77: 2578–2586

79 Sitati E M, Diamond M S. $CD4^+$ T-cell responses are required for clearance of West Nile virus from the central nervous system. J Virol, 2006, 80: 12060–12069

80 Shrestha B, Diamond M S. Role of $CD8^+$ T cells in control of West Nile virus infection. J Virol, 2004, 78: 8312–8321

81 Brien J D, Uhrlaub J L, Hirsch A, et al. Key role of T cell defects in age-related vulnerability to West Nile virus. J Exp Med, 2009, 206: 2735–2745

82 Croft D R, Sotir M J, Williams C J, et al. Occupational risks during a monkeypox outbreak, Wisconsin, 2003. Emerg Infect Dis, 2007, 13: 1150–1157

83 Huhn G D, Chase R A, Dworkin M S. Monkeypox in the Western hemisphere. N Engl J Med, 2004, 350: 1790–1791

84 Fenner F. Mousepox (infectious ectromelia): past, present, and future. Lab Anim Sci, 1981, 31: 553–559

85 Fang M, Cheng H, Dai Z, et al. Immunization with a single extracellular enveloped virus protein produced in bacteria provides partial protection from a lethal orthopoxvirus infection in a natural host. Virology, 2006, 345: 231–243

86 Karupiah G, Buller R M, Van Rooijen N, et al. Different roles for $CD4^+$ and $CD8^+$ T lymphocytes and macrophage subsets in the control of a generalized virus infection. J Virol, 1996, 70: 8301–8309

87 Fang M, Lanier L L, Sigal L J. A role for NKG2D in NK cell-mediated resistance to poxvirus disease. PLoS Pathog, 2008, 4: e30

88 Fang M, Sigal L J. Antibodies and $CD8^+$ T cells are complementary and essential for natural resistance to a highly lethal cytopathic virus. J Immunol, 2005, 175: 6829–6836

89 Parker A K, Parker S, Yokoyama W M, et al. Induction of natural killer cell responses by ectromelia virus controls infection. J Virol, 2007, 81: 4070–4079

90 Fang M, Sigal L J. Direct CD28 costimulation is required for $CD8^+$ T cell-mediated resistance to an acute viral disease in a natural host. J Immunol, 2006, 177: 8027–8036

91 Fang M, Orr M T, Spee P, et al. CD94 is essential for NK cell-mediated resistance to a lethal viral disease. Immunity, 2011, 34: 579–589

92 Fang M, Roscoe F, Sigal L J. Age-dependent susceptibility to a viral disease due to decreased natural killer cell numbers and trafficking. J Exp Med, 2010, 207: 2369–2381

93 Xu R H, Fang M, Klein-Szanto A, et al. Memory $CD8^+$ T cells are gatekeepers of the lymph node draining the site of viral infection. Proc Natl Acad Sci USA, 2007, 104: 10992–10997

94 Kastenmuller W, Torabi-Parizi P, Subramanian N, et al. A spatially-organized multicellular innate immune response in lymph nodes limits systemic pathogen spread. Cell, 2012, 150: 1235–1248

**Open Access** This article is distributed under the terms of the Creative Commons Attribution License which permits any use, distribution, and reproduction in any medium, provided the original author(s) and source are credited.
